# *“I’m Not Right to Drive, but I Drove out the Gate”:* Personal and Contextual Factors Affecting Truck Driver Fatigue Compliance

**DOI:** 10.3390/ijerph22111724

**Published:** 2025-11-14

**Authors:** Gregory J. Casey, Toby Miles-Johnson, Garry J. Stevens

**Affiliations:** 1 School of Social Sciences—Criminology and Policing, Western Sydney University, Liverpool, NSW 2170, Australia; t.miles-johnson@westernsydney.edu.au; 2 School of Social Sciences—Humanitarian and Development Research Initiative (HADRI), Western Sydney University, Penrith, NSW 2751, Australia; g.stevens@westernsydney.edu.au

**Keywords:** fatigue, truck driver, public health, risk, heavy vehicle national law, compliance, theory of planned behaviour

## Abstract

Truck drivers experience an elevated risk of being involved in a fatigue-related crash or incident. In Australia, approximately one third of fatal truck crashes are fatigue-related. Various contextual factors are known to increase truck crash risk, including long working hours, irregular schedules, delays while loading and unloading and limited access to suitable rest areas. Studies investigating personal factors affecting Australian truck drivers’ attitudes and compliance with fatigue-management requirements, however, are lacking. Semi-structured interviews were conducted with Australian truck drivers and transport managers (N = 44) to determine how personal and contextual factors influence their intention to comply with fatigue regulation. The findings indicate that personal factors such as familial pressure, financial viability as well as inflexible enforcement and its personal consequences may influence fatigue-related health risks and compliance behaviours. This includes contextual factors such as work scheduling, training and new risk monitoring technologies. It is argued that government, transport industry peak bodies, managers, unions and truck drivers should work together to co-develop fatigue management strategies that account for personal factors likely to influence truck drivers’ intentions regarding fatigue compliance. This will support them to engage in safer and healthier fatigue management practices.

## 1. Introduction

Fatigue-related road crash risks are often associated with truck driving [1,2,3,4]. Fatigue can be attributed to a range of circadian, homeostatic and task-related factors [5]. Casey, Miles-Johnson and Stevens [6] found that fatigue risks are heightened in occupational truck driving because these workers spend extended periods of time driving and are subject to sleep deprivation, night shift work and irregular schedules. These are typical occupational conditions affecting transport industries both in Australia and globally [3,7,8]. As a result, truck drivers are often limited in their ability to obtain “recuperative rest”, i.e. rest that enables recovery from fatigue and sleepiness [5] (p. 499), thus increasing their risk of driving while fatigued [9]. The association between truck driver fatigue (TDF) and truck crashes is widely reported in research, road crash data, police reports and media articles [3,4,5,7,10]. This is because truck driving generally occurs on public roads and when truck drivers experience fatigue they pose a health and safety risk to themselves and the public.

In Australia, road crash deaths and injuries are recognised as a major public health problem and it is estimated that fatigue is a factor in one in three fatal truck crashes [4]. The social cost burden they impose includes fatalities, injuries and substantial property and administrative costs [10]. When fatigue-related truck crashes occur, they impose health-related burdens as well as personal and indirect financial costs, and a likelihood of human injury and death [9]. For example, in Australia, between 2015 and 2024, more than 1600 people were killed in crashes involving heavy trucks—defined as a truck with a GVM of over 4.5 tonnes [11]. At a broader level, the social costs are estimated at AUD 3.2 million for each road fatality (including truck related crashes) [10] with the annual cost of fatigue related road crashes for all road users totalling AUD 3 billion [9].

To reduce the public health risks associated with TDF, Australia, like other countries, applies restrictions on the number of hours truck drivers may work and specifies the number of hours they must rest [12,13,14]. However, rest regulations are only effective in reducing road crashes if they are obeyed [15]. Previous studies have explored the influence of workplace factors on truck drivers’ responses to fatigue compliance [6,12,16,17,18] and on their personal physical and mental health [19,20,21]. In Australia, the risk of TDF is exacerbated by workplace contextual factors such as different freight types and the need to move freight across vast distances. This also includes unfavourable weather conditions, traffic delays, delays in relation to the loading or unloading of freight, limited access to suitable rest-stops and commercial pressures for expedited delivery [6,12,16,22].

When working under these conditions, drivers may decide (or be encouraged by management) to skip rest breaks and mask this behaviour. Masking, for example, may include recording freight-related waiting or loading time (which is ”work” time) as “rest” time in their work diaries [17]. In addition, truck driving is typically a sole activity, meaning that fatigued drivers do not have the option of being relieved by a co-driver, thereby reducing their ability to rest [20]. Scheduling demands may encourage risk-related practices such as exceeding speed limits, working excessive hours (to make up for delays), or using drugs to suppress the onset of fatigue [16]. Remuneration type may also affect risk-related practices and freight delivery. This is because approximately two-thirds of Australian truck drivers are paid by the number of kilometres they travel or the tonnage of freight they deliver [18]. Although this may have positive commercial outcomes for freight delivery it is likely to increase driver fatigue risks. This is because drivers paid this way are not being paid when they are not driving [23].

TDF risks may be affected by other contextual factors such as disparities regarding TDF management policies. It may also be affected by differences in legislative structures across Australian states that can result in fragmented TDF arrangements [12,24]. This is likely to increase fatigue risks because some drivers may not be aware of respective changes to TDF management systems. This may include differences in Australian transport industry operations that change in relation to regulatory guidelines as drivers cross different jurisdictions whilst working [12,24,25].

While there is a body of literature that analyses TDF [9,26,27,28,29,30,31,32], Phillips, Kecklund, Anund and Sallinen [33] state that a gap remains between transport-related fatigue research and fatigue management practice. They argue that *“Further research is required to explain this translational gap, to investigate why measures are not implemented and if they are implemented, whether implementation is optimal”* [33] (p. 759). The available research has focused on organisational, logistics and enforcement factors affecting TDF, but there have been few studies exploring whether personal factors influence truck drivers’ and transport managers’ intentions regarding fatigue compliance. These are lacking in both the Australian and international literature [9]. This includes family or personal financial concerns and how these may affect fatigue perceptions and compliance.

Attitudes towards risk-taking regarding compliance may be influenced by family, culture, friends, emotions, mental and physical health. They may also be influenced past experiences, motivation, perception and interpretation of risk [34,35]. In this sense, personal factors and related concerns will shape how an individual perceives and interprets situations and responds to their environment [34]. The response behaviour, therefore, is a complex interplay between personal factors and the contextual environment in which they occur [35].

To extend on the work of Ren et al. [9] and better understand how personal and contextual factors influence the intentions of Australian truck drivers’ and transport managers’ compliance with fatigue laws, Ajzen’s [36] Theory of Planned Behaviour (TPB) was applied as a theoretical framework for this study. The TPB has been used widely for understanding and predicting behaviour [36,37]. It has been applied in a diverse range of studies analysing behaviour, such as physical activity [38], crime reporting [39] and truck driver safety in the USA [40]. Thiffault [41] recommended the use of the TPB to evaluate the determinants of risk-related truck driving behaviour in Canada. In that study, the TPB was applied as a theoretical framework to develop driver-oriented interventions to manage occupational risk.

**Figure 1:**
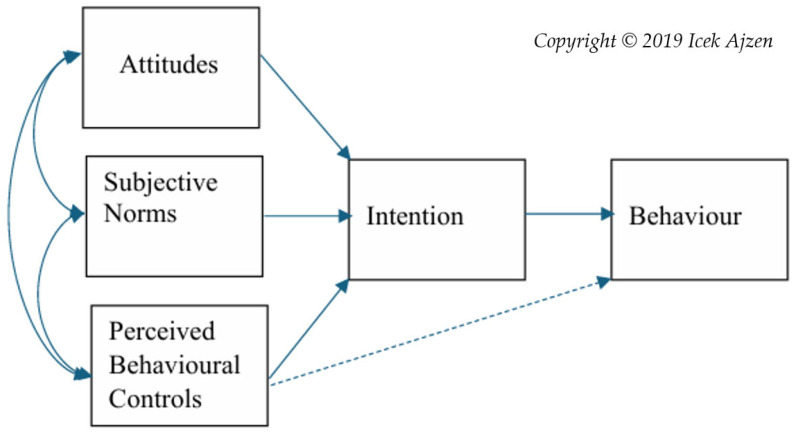
The Theory of Planned Behaviour.

While studies applying the TPB to examine truck driving behaviour in Australia are limited, its use in this study enabled us to identify specific driving-related tasks associated with risk. This includes analysing pivotal choices regarding fatigue management and compliance. By applying components of the TPB (Attitude, Subjective Norm and Perceived Behavioural Control), one can determine intention to behave a certain way, which is critically shaped by a person’s attitude, and considers their disposition to respond favourably to an object, person, institution or event [36] (see Figure 1). Within Figure 1 solid lines represent a direct relationship between factors, while the dotted line reflects an inferred relationship between perceived and actual behavioural control and behaviour [36].

As stated, contextual factors shape truck driving experiences and TDF compliance [16,18,22]. This includes social pressures or subjective norms, and a person’s sense of self-efficacy or perceived behavioural controls regarding their capacity to perform an indicated behaviour. It was determined, therefore, that each of these components of the theory would enable a better understanding of contextual pressures that shape driver perceptions of fatigue risk. In addition, an analysis of perceived behavioural controls would enable the research team to identify specific personal influences that may shape truck drivers’ TDF compliance and perceptions of risk. It would also facilitate an analysis of driver fatigue risks when personal and contextual demands conflict.

Accordingly, the research presented is a qualitative study that is both exploratory and inductive. It analyses a phenomenon that is not clearly understood in Australian contexts and builds insights from the collected data by applying (rather than testing) an established theory. Inductive research does not begin with a hypothesis; it is often guided by research questions containing “How may”. These types of questions are often exploratory and seek to understand potential possibilities or impacts [42]. Given that the study would be exploratory, it was determined that the following research questions would be appropriate to guide the research. (1) How may personal factors influence truck drivers’ attitudes, intentions and behaviours regarding TDF-related risks? (2) How may risk perception change when personal and contextual situations conflict and how does this affect TDF law compliance? And (3) How may the influence of transport managers shape truck drivers’ perceptions of TDF risk and law compliance?

## 2. Methods

### 2.1. Site Selection

To answer the three research questions, a purposive sampling approach was used to recruit truck drivers and transport managers working in long haul truck operations. This is because it was determined that these individuals were most likely to provide an informed discussion regarding the research questions. Following Western Sydney University Ethics Committee approval (Approval No H15319), three large transport companies and three representative organisations (a trade union and two employee representation bodies) were approached about participation in this study. To access a sufficient sample of people employed across the Australian transport, postal and warehousing sectors and to include participants working across all Australian states and territories, it was anticipated that the six transport industry organisations would be able to provide access to a representative sample of participants.

### 2.2. Recruitment Process

The final sample of participants were provided by five of the six organisations as one organisation declined to participate. Each participating company and organisation was provided with information about the research aims and processes, and once agreement to participate was reached, a recruitment text was sent by each organisation to their respective employees. The texts (see Appendix A) invited truck drivers and managers working in long haul operations (the inclusion/exclusion criteria) to participate in an interview regarding TDF. As the recruitment process began (and employees from the organisations contacted the primary researcher to participate in this study), two industry podcasts discussed the research.

Whilst it is unknown how many listeners tuned into the podcasts, it is important to acknowledge that the additional publicity afforded to the research was likely to have affected the recruitment process. This is because following each podcast the principal researcher was contacted by additional participants volunteering to be interviewed. Due to the lack of information regarding how the participants were informed about the study, response rates could not be calculated. This was also affected by each organisation facilitating (via their own internal processes) initial recruitment. Once the final sample of participants was confirmed, telephone or videoconference interviews were scheduled based upon participant convenience. Each participant was allocated a pseudonym, and all participant data was deidentified. Forty-three interviews were conducted by telephone, and one interview was conducted via video conference. Once verbal consent was obtained all interviews were audio recorded.

### 2.3. Participants

A total of 36 truck drivers (New South Wales (n = 7), Queensland (n = 14), Northern Territory (n = 1), Victoria (n = 5) and Western Australia (n =9)) and eight transport managers were included in this study (total N = 44). Participants’ ages ranged from 24 to 77 years. Their workplace experience ranged from 1 to 55 years. The sample included six truck drivers who identified as women. This is also representative of the proportion of women (18%) working in the Australian road transport sector [43]. The sample of managers comprised men only because no women transport managers contacted the researcher. The participant characteristics are shown in Table 1. The geographical locations of the truck drivers are shown in Table 2.

The participants based in states where the Heavy Vehicle National Law (HVNL) applies comprised 72% of the sample, while those working in states where TDF is managed via Workplace Health and Safety (WHS) laws comprised 28%. This is proportionate to the total number of truck drivers identified as working in HVNL states (81.5%) and in WHS states (18.5%), with the proportions determined from compiled data. Jurisdiction types and the total number of drivers in this study that work in each are shown in Table 3.

Because all managers are responsible for truck drivers working across all Australian jurisdictions (both HVNL and WHS) and as such typically travel between different depots in geographical locations, the specific site where they were positioned was not recorded at the time of interview.

### 2.4. Semi-Structured Interview Measures

It was determined that semi-structured interviews offered the best means to elicit a deep understanding of participant experiences of fatigue-related phenomena [44]. Accordingly, separate interview schedules were prepared for each cohort to reflect the separate roles of truck drivers and transport managers. Once ten interviews had been conducted, it was determined that the interview questions contained in each schedule were validated. This is because the responses were checked for clarity, consistency and the ability to collect the intended information. In addition, the nature of the semi-structured interviews enabled the researcher flexibility in terms of responses to the participants answers and based on the flow of the interview (and information shared), follow-up and the prompt questions that were posed. This process also validated the interview questions [44].

To understand how contextual pressures shape driver perceptions of fatigue risk, the interview questions were arranged in sections consistent with the core components of the TPB (beliefs and attitudes, subjective norms and behavioural controls). Examples of common questions relating to the attitudes amongst both cohorts (truck drivers and managers) included *”In your opinion, is truck driver fatigue an important issue?”*. Questions regarding subjective norms included *“In your opinion, what are the strongest influences on [truck drivers/managers] perceptions of truck driver fatigue?”*. Questions regarding perceived behavioural controls included *”What kind of things might change a [truck drivers/managers] intentions about complying with truck driver fatigue laws during a shift?*”. A screening question determined each participant’s awareness of the physical effects of TDF, with an alternate set of questions prepared should participants indicate a lack of awareness. However, these were not used as all participants indicated an of awareness of TDF. The full interview schedule is provided in Appendix B and Appendix C. Both appendices include the coding schema (from Section 3) regarding interview items relating to the components of the TPB for driver and manager interviews (see subheading *Coding Schema—Interview Items and TPB Components*).

Interviews continued until the primary researcher was satisfied that no new information was forthcoming. Saturation was considered reached in consultation with the other members of the research team. On completion of the interviews, each audio recording was transcribed and identifying information was removed. The interviewer (author one) remained conscious of potential gender and power imbalances when interviewing women truck drivers, and as a male, was conscious of differing social processes, context and meaning that may affect women participants in their work environment [45,46]. To minimise the effect of any of these potential imbalances, the researcher deliberately encouraged women truck drivers to discuss power imbalances that may intrinsically affect them.

### 2.5. Data Analysis

The data analysis was conducted using thematic analysis. This is because of its capacity to enable an in-depth exploration of phenomena with a limited theoretical and empirical background [47]. To minimise potential researcher bias, Braun and Clarke’s [47] analytical method was applied. This involved ensuring familiarisation with the content by reviewing the interview transcripts several times. Following this process, initial codes were generated in the context of the research questions, the components of the TPB (attitude, subjective norms, and behavioural controls) and risk-related material. In addition, an inductive approach was used to search for themes that were induced from the codes. An iterative review process was then employed, whereby repeated referral to transcripts and the literature ensured the validity of the produced themes [48]. This process enabled the researchers to rigorously determine the thematic saturation point and to determine that the data fit into the existing framework of the TPB. To complete this process, the research team recorded and mapped each theme and consulted on the initial findings. This prompted further review and refinements, and the definition and naming of the final overarching themes. In addition, the research team reciprocated roles as the primary data coder and co-raters. This ensured consistency and acted as a validity check. Following the iterative review process, it was clear that no new themes or new meaningful patterns could be identified. The research team, therefore, was confident that the themes identified were well developed and captured the essence of the dataset.

## 3. Results

All the participants were able to discuss TDF in depth. This increased confidence that the participants could answer the research questions in an informed way. When asked if fatigue was an issue within the transport industry, all (n = 44/44) participants were unequivocal in their responses, *“Oh in my opinion it’s the main thing”* (Jane, truck driver, 35 years old), *“Bloody oath. Oh God yeah.”* (Nathan, truck driver, 58 years old). Personal experience enabled the participants to recognise fatigue in themselves and others, with eight specifying that they identified their own fatigue when they became irritable, started yawning or struggled to change gears or maintain their position in their driving lane. Five participants who were transport managers reported that they identified TDF in truck drivers when they noticed changes in mood swings, incomplete paperwork, appearance or tardiness.

The risks associated with TDF were raised more than 130 times throughout the interviews. While fatigue-related crashes were the most common risk context discussed, participants frequently discussed personal challenges, such as financial losses and viability, and risks associated with TDF enforcement-related sanctions. Many participants also described positive contextual factors relating to organisational practices that reduced TDF-associated risks. An analysis of the findings in relation to fatigue risk identified three themes and four sub-themes:

(1) Personal factors influence TDF risk and compliance behaviour.
  (a) Personal financial viability and payment methods influence TDF risk behaviour.
  (b) Fatigue risks for owner-operators and small companies.
(2) Inflexible enforcement and financial impacts affect fatigue.
(3) Organisational practices affect TDF risks.
  (a) Electronic monitoring may reduce TDF risks.
  (b) Fixed roadside monitoring cameras can increase TDF risk.

### 3.1. Personal Factors Influence TDF Risk and Compliance Behaviour

This study identified many factors that go beyond the well-described work-related TDF risk factors [3,6,16,49]. Most participants (n = 28/44) discussed that before leaving home, family concerns influenced their TDF risk responses. For example, some participants spoke about their ”sleep quality” being impacted when children or partners were sick or distressed. This meant they experienced pre-shift fatigue.

*“I’m fatigued, I’m not right to drive, but I still showed up for work, I still started the truck and still drove out the gate”*

(Rowan, truck driver, 49),

Performance capability, and TDF crash risk, therefore, is likely to be affected before they start work [5]. When pre-shift fatigue occurs, however, drivers can remain compliant with prescriptive work and rest hours because the definition of “rest” only requires disengagement from work involving a truck with a gross vehicle mass (GVM) of greater than 12 tonnes [14] *(Sections 7 and 221)*. It does not mean that periods of *“recuperative rest”* [5] (p. 499) have occurred. Amongst those who work on the premise that *“legal equals safe”*, driving in conditions where dangerously low levels of rest have occurred increases the likelihood that such behaviour will be normalised [50] (p. 356).

Personal factors were repeatedly raised as an important influence on drivers’ TDF responses. For example, if a driver fell ill while on a long-distance journey, they often lacked *“access to medical services or even a chemist [pharmacy]”* (Oscar, driver, 59). In addition, the size of their truck precluded them from driving to, or parking near, such services. This was reported as often resulting in untreated illness which led to greater fatigue.

Many participants (n = 23/44) spoke of being pressured to immediately return home when family emergencies or events arise, such as birthdays, school or sporting events, or social gatherings. Some participants articulated the impossibility of this demand when work-related factors (such as being redirected to a new destination) prevent this. Some participants discussed the emotional pressure this creates and how the effects of such pressure could be acute. *“When you let them down, they don’t fully understand, they can say, well you don’t care, or you’re just useless”* (Dominic, driver, 27). Participants talked about how this pressure placed drivers at risk of their *“home life breaking up”* (Belinda, driver, 48).

Almost half of the participants (n = 20/44) discussed that when personal and job-related demands conflict, it can strain relationships, as well as increase estrangement and the likelihood of marriage breakdown. It can also create isolation with family and friends. It was discussed that this emotional and relationship pressure increases stress and the likelihood of distraction [51], which undermines drivers’ mental health [21] and can lead to detrimental road safety outcomes [52]. This was associated with fatigue because under these circumstances rest and sleep is more difficult to obtain. *“So, you get that emotional drain, which is fatiguing, then that starts the mental drain, then it starts the physical drain”* (Darcy, driver, 61).

Eight participants discussed how domestic arguments (whilst working far from home) influence their intention to not comply with TDF regulations. This is because these situations will often *“play on their mind and contribute to fatigue, stress, anxiety”* (Jane, driver, 35). It is also likely to increase their desire to *“hurry to get home so that they can try and fix it up”* (Ursula, driver, 57). Notably, one third of participants (n = 16/44) suggested that truck drivers often prioritise family or personal obligations over workplace compliance demands. For example, to resolve domestic disputes and return home quickly some drivers react by *“push[ing] hard to get where he needs to be to keep everything sweet”* (Dominic, driver, 27). Yet, this action is likely to increase TDF-related crash and compliance risks.

One participant referenced a colleague who drove for *“20 h straight”* to get home for a school presentation (Lachie, driver, 56). To understand this in context, driving for 20 h straight means the driver must forgo all mandated rest periods during the shift (for example, 4 × 15 min rest breaks or 2 × 30 min rest breaks) and a 7 h continuous stationary rest break. Furthermore, if this occurred at the end of the drivers’ working week, it could mean they forgo a mandated 24 h rest that is required within each 7-day period [53] *(Schedule 1 and 2)*. Driving for 20 h straight, therefore, could enable the driver to arrive home up to 24 h earlier than compliance with TDF laws would allow, yet this would increase the risk of a fatigue-related crash or sanctions.

When such challenges were discussed with managers, some spoke of offering help to drivers’ facing personal or family related issues, such as allocating additional time off work. Whilst this type of action was likely to support drivers, it was suggested that these situations were unlikely to occur due to a reluctance among drivers to discuss fatigue issues [6]. It was also affected by fear of disclosure and a resultant job loss,

*“So long as they make that phone call [to discuss personal issues affecting fatigue], you can manage it. But if they fear for their job, they’re going to come to work and put everyone at risk, including themselves”*

(Harry, transport manager, 55 years old).

Following this discussion, it was determined that the capacity of managers to institute a culture of safety within their company is likely to vary depending upon the resources available to them. This is because smaller operators often lack the resources and skills necessary to promote such procedures [54].

#### 3.1.1. Personal Financial Viability and Payment Methods Influence TDF Risk Behaviour

Multiple participants (n = 17/44) spoke of how drivers’ responses to TDF can be influenced by concerns about personal financial viability. This is significantly influenced by how drivers are paid. Payment methods discussed included hourly rates, and *“incentive payments”* [18] (p. 27), such as those structured on a per-kilometre or per-trip basis. For example, 67% of truck driver participants identified as being paid by incentive payments. Payment methods received by drivers are shown in Table 4.

For drivers on incentive payments, delays arising from waiting to load or unload freight, as well as traffic hold-ups or taking rest breaks (for which they are not paid) could mean that their next freight load is missed. As a corollary, they are penalised financially, missing out on additional payments of *“500 bucks, because you’ve got no load”* (Lachie, driver, 56). The type of load carried by truck drivers was frequently discussed in the research. For example, general freight was identified as being the most common freight type carried by the participant cohort. Load types are shown in Table 5.

When financial concerns were discussed, participants indicated that drivers are more likely to breach TDF laws and *“do what they gotta do to put food on the table”* (John, driver, 37). In addition, it was clear that freight loading delay accentuates the inequity of incentive payments. For example, one participant said the following:

*“Drivers who are on hourly rates take it all in their stride because they’re still getting paid. Guys who are on trip money, who have to wait for two hours while everybody dicks you around, is technically not getting paid”*

(Warren, driver, 65).

From these discussions, it was reasonable to surmise that drivers being paid per km or per trip may be less disposed to stop and rest when they are tired. This is because *“if the truck’s not moving, you’re not earning money”* (John, driver, 37). This type of attitude is likely to increase the risk of TDF-related incidents.

#### 3.1.2. Fatigue Risks for Owner-Drivers and Small Companies

Financial pressures and incentives were also discussed in the context of truck drivers working as ”owner-drivers” or sole traders/contractors who use their own truck to move freight. Half the participants (n = 22/44) talked about the economic pressures owner-drivers face, which increases the likelihood of TDF risk behaviours. This is because of the financial demands of running their own business. To maintain business viability, eight participants expressed concerns that owner-drivers were more likely to prioritise financial returns over good TDF practice. Some noted that current low payments for freight delivery further exacerbated these pressures.

*“They can’t just do Sydney [to] Melbourne [900 km] twice a week, they’ve actually got to do it five or six times to be able to pay themselves a reasonable wage because the rate of the freight has been screwed down that much”*

(Lachie, driver, 56).

One participant suggested that remunerating drivers for freight-related waiting and loading times could lead to *“a different ball game”* (Ryan, manager, 69). This is because it could incentivise transport customers to minimise freight loading or unloading delays, thereby relieving income-related stress for drivers on incentive payments. While the finding that payment methods adversely influence TDF risk behaviour is not new [17,18], it indicates that this issue is an ongoing problem in relation to TDF risk.

### 3.2. Inflexible Enforcement and Financial Impacts Affect Fatigue

Most participants (n = 27/44) acknowledged that TDF laws must be enforced but expressed concern about the lack of flexibility regarding contextual factors affecting driving. The concerns expressed included that TDF laws were constructed under a *“One size fits all”* approach (Gerard, driver, 70). Many participants were concerned that the TDF laws do not support the variable nature of the freight loads that trucks carry [12]. One participant related the lack of flexibility to how

*“The rules don’t coincide with the human experience, which is individual to every person”*

(Vicki, driver, 49).

This description supports research by Miller et al. [55] whose study identified a need for individual rather than generic interventions regarding fatigue. Several participants (n = 14/44) explained that if they complied with prescribed work or rest requirements, taking a rest break could, perversely, be detrimental to their ability to manage TDF. If they did not feel fatigued, they were unable to rest, but if they felt fatigued at a later point in their shift, they could not stop and rest due to diminishing available work hours. Respondents felt this was problematic. Resting only when they felt fatigued risked breaching prescriptive laws, thereby increasing the likelihood that they would be penalised under enforcement actions.

Most participants (n = 30/44) were concerned by this in relation to the likelihood of receiving expensive fines or financial penalties for not taking prescribed rest. In detailing this, some participants spoke about fines costing the drivers as much as *“the weeks’ pay”* (Andrew, driver, 29), which could put personal relationships at risk. This is because when a driver’s income needs to be redirected from the household budget or savings to pay a fine it *“reflect[s] back on the family, the innocent people”* (Kevin, driver, 67). Six participants spoke of how such penalties may affect drivers if they are fined, because they are often distracted by worry regarding how to *“pay the bills”* (Peter, driver, 66). Consequently, this causes drivers to experience stress, leaving them “*stressing while they’re driving a 68-tonne truck”* (Kevin, driver, 67). It was evident from the interviews that receiving fines due to problems complying with prescribed rest periods generated substantial stress for drivers that affects their concentration and detracts from safe driving.

Five participants reported that court processes in relation to fines and penalties (and related disputes) often increased stress for drivers. This is because, to challenge a fine, drivers must stop work and travel to the court nearest to where the fine was issued. This process must occur on a date nominated by the court, which many drivers perceive (regardless of the outcome) as being unfair. In addition, the driver is liable for travel and accommodation costs, legal fees and the loss of one or more days of work. These combined costs often exceed the original penalty, thereby resulting in greater financial hardship. One participant said this means *“The process becomes the punishment”* (Matthew, driver, 60) and for this reason *“a lot of blokes won’t contest fines”* (Oscar, driver, 59).

Truck drivers typically bear collective responsibility for fatigue law breaches within the Australian transport industry [17,56]. This is despite their limited control over numerous factors. Perceptions of fairness regarding rest compliance and the reality of having to do so at prescribed times is, therefore, likely influenced by driver-focused enforcement, which is a recognised phenomenon [22,25]. However, components of prescriptive TDF laws enable penalties for other parties in the ”chain of responsibility”, that is, managers, schedulers, consignors or others who hold TDF management responsibilities [14] (Section 5), but these components of prescriptive TDF laws are rarely enforced [17,25,56,57]. This was reflected in participant comments indicating that driver-focused enforcement persists, and that this contributes to rest-related stress for drivers, *“Because the person at the coalface is the driver, and they’re the ones who bear the brunt of it”* (Dominic, driver, 27). When chain of responsibility and rest compliance were discussed with managers, most managers (n = 6/8) said that enforcement activity was rarely directed towards them. As one manager implied, managers are more likely to be influenced by *“that risk and reward thing”* (i.e., company scheduling imperatives) rather than TDF laws. The manager explained that,

*“You send 10 trucks down the road overloaded and with tired drivers in them. How many are gonna get caught? Probably only if something goes wrong”*

(Aaron, manager, 62).

How TDF laws are enforced is important because if the procedures regarding regulation are not perceived as legitimate, it is likely that drivers will not comply with the rules [58,59]. As one participant commented, *“If you recognise the law is justified and its fair and reasonable, you are more likely to comply with the law”* (Peter, driver, 66). Perceived unfairness regarding the current enforcement of TDF laws and legal proceedings may, therefore, have implications for drivers’ attitudes, intentions and compliance with fatigue regulations and the related risk of being involved in a crash.

### 3.3. Organisational Practices Affect TDF Risk

Most participants (n = 40/44) spoke of organisational practices and contextual factors which they felt contributed positively to reducing drivers’ individual TDF risks. This included comprehensive company induction and training programs that enabled drivers to *“understand exactly where you stood”* (Matthew, driver, 60), regarding TDF management and company policies. Just over half of the participants (n = 24/44) spoke of how experience and knowledge of TDF laws equipped them to manage fatigue. They also spoke about how, this enabled them to challenge their manager regarding fatigue compliance without risk of job loss, when necessary. If they felt the need to stop and rest during a shift one participant said,

*“You felt brave enough to say to your boss, I can’t do that cause I got to have a 7 h break. And thought OK, well maybe these fatigue laws work*.”

(David, driver, 58)

In addition, organisational practices that included flexible delivery schedules were identified by 25 participants as enabling better TDF management. This is because flexible schedules removed additional pressure on drivers if they were delayed or if they needed to stop and rest. *“So, if you need to pull up, then that’s what you do”* (John, driver, 37). This finding reflects the work of Tucker and Folkard [60] who suggest that fatigue management is likely to be improved when the timing of rest is at the discretion of the individual. Some participants (n = 15/44) positively discussed organisational practices regarding TDF compliance. This included companies providing well-maintained trucks that were equipped with rest-related features such as comfortable bedding that enables restful sleep.

Five managers contextualised positive organisational practices in relation to companies providing drivers with time away from work to fully recover from fatigue without it affecting job security. One manager spoke of a company psychologist being *“on stand-by”* (Brent, manager, 41) to assist employees when required. Some participants spoke of positive organisational practices only being offered by larger companies. This is because for smaller companies, *“it’s a totally different thing”* (Matthew, driver, 60). Interestingly, research suggests that smaller companies often lack human, physical and financial resources to provide initiatives and resources that may enable or facilitate rest and reduce TDF risk [54].

#### 3.3.1. Electronic Monitoring May Reduce TDF Risks

Research suggests that when transport organisations provide drivers with electronic monitoring devices (e-monitors) it can assist drivers to maintain good driving practices. Gruchmann and Jazairy [61] (p. 326) argue that further research, however, is needed regarding the different types of assistance systems, *“particularly those that blur the line between support and oversight”*. When discussing organisational practices such as the distribution of e-monitors or electronic work diaries (e-diaries) to reduce TDF risk, 33 participants in this study discussed how e-monitors and e-diaries may positively influence rest and compliance behaviour. When talking about e-monitors, which include eye and speed monitoring devices, telematic data systems, GPS data tracking and e-diaries, none of the participants spoke about privacy concerns regarding their use (particularly those distributed or mandated for use by companies). According to Gruchmann and Jazairy [61], this may be due to participants’ awareness of TDF-related risks and evaluating a lack of privacy versus potential safety. It may also be due to the compliance benefits that such devices may offer. For example, participants described how e-diaries provide audible and visual warnings regarding forthcoming required rest breaks. Drivers input work and rest hours but thereafter the system works automatically, reducing the administrative burden and the risk of clerical error.

*“I love it. It’s so easy, so simple, it tells you what you can and can’t do. The only trouble is you can’t cheat it. With an electronic logbook, you’re out of hours, you stop. With a paper book, I’ll just drive to my destination then just manipulate the driving time.”*

(Oscar, driver, 59).

Over half of the participants (n = 28/44) spoke positively of e-monitors in relation to a reduction of TDF risk. This is because they perceived that managers could monitor TDF in real time to support drivers. One driver appreciated that such monitoring could also *“hold the company to account”* (Tom, driver, 35). This is because managers who pressure drivers to engage in non-compliant rest behaviours were more likely to be exposed. Eight participants highlighted that while the benefits of organisational practices such as the distribution of e-monitors could reduce TDF risks, their usage is generally limited to larger freight companies which have the human resources needed to monitor live data [54].

#### 3.3.2. Fixed Roadside Monitoring Cameras Can Increase TDF Risk

Despite strong support for ”in-house” TDF monitoring practices, the attitudes towards systems managed by enforcement organisations differed sharply. For example, 23 participants argued against the use of one monitoring device: the National Safety Camera Network [62]. This camera network is a series of fixed roadside monitoring cameras placed along highways in states where prescriptive TDF laws apply. The cameras record the location and direction of a truck and the time it passes from one camera to another. Camera data may then be used for compliance purposes to calculate breaches of TDF regulations and prosecute drivers [62].

Eight participants spoke about the negative effect such cameras have on drivers and on TDF compliance. Critiques were linked to camera monitoring in relation to the mandatory continuous 7 h rest breaks that are required within each 24 h period. Problems were attributed to the challenge this causes for a fatigued driver if they stop earlier within that period to rest for several hours. In this instance, camera data calculations (determining time and distance travelled) would show that insufficient time remained for that driver to take a 7 h *continuous* rest break within the 24 h period. This was thought to likely expose the driver to the risk of sanctions regarding non-compliance with TDF laws. Consequently, fatigued drivers may

*“push themselves to get past these cameras to have a [mandatory 7 h continuous stationary] rest on the other side, [which] is a dangerous thing”*

(Oscar, driver, 59).

The perception that fixed roadside monitoring cameras are forcing drivers to drive while fatigued is likely linked to a lack of flexibility regarding the prescriptive 7 h rest break. Because these cameras may negatively influence drivers’ attitudes, intentions and behaviours regarding TDF compliance and the associated risks, this is an important new finding regarding TDF risk-related behaviour.

## 4. Discussion

The findings from this study reveal several personal factors that influence Australian truck drivers’ and transport managers’ intentions to comply with fatigue regulation and how they manage health and contextual risks relating to fatigue. For example, long-distance drivers whose sleep is disrupted at home because of family-related issues may spend an entire shift at risk of experiencing fatigue. This finding provides empirical context to previous research by Feyer and Williamson [63] and Dawson and Thomas [64], who suggest that prior sleep can predict fatigue. This is because the impact of pre-shift fatigue on driving performance is likely to increase risk despite compliance with the prescribed hours of work and rest.

In addition, because long periods are spent away from home, drivers are less likely to be able to manage personal or family responsibilities or be able to participate in family or social activities [65]. Subsequently, fatigue compliance was seen to act as a contributing stressor with participants often reporting that family requests are difficult to achieve without forgoing rest or breaching TDF regulations. These challenges were discussed further in relation to personal factors and related stress. These were discussed in terms of the potential for additional emotional pressure and strained relationships, the risks of potential marriage breakdown and estrangement from family and how this affects driver’s stress. This also included discussions about anxiety, mental and physical health and fatigue. While it was clear that familial circumstances have the potential to disrupt sleep and increase driving risks, families are likely unaware of the specific risks this may pose for their loved ones. Despite previous calls for education and support programs for truck drivers’ families [66,67], no such programs currently exist in Australia [68]. Other occupational groups, such as the Australian military, have co-developed online modules to support partners and children and increase understanding of ”work away”, role and health requirements [69]. Such programs could potentially inform similar initiatives in the transport sector.

To manage or avoid strained personal relationships, the drivers in this study reported being forced to choose between breaching TDF laws (thereby avoiding rest breaks and exceeding daily work limits) and returning home several hours earlier or complying with TDF laws and returning home later. This is problematic, because depending on their choice, drivers prioritise family relationships over the risk of a fatigue-related crash or the risk of incurring fines. This suggests that personal circumstances are likely to be a predictive factor regarding TDF-related crash risks.

According to the TPB, the antecedent of an attitude is determined by beliefs that associate a behaviour with a certain outcome. Attitude is also determined by the strength of the belief that the behaviour will result in the preferred outcome under consideration. Ajzen [36] argues that beliefs may be formed from direct observation (for example, a previous relationship breakdown), by self-generated inference processes (working away from home for long periods is likely to harm relationships) or by accepting information from other sources (colleagues who have experienced relationship breakdown). In context, the TPB suggests if a driver evaluates personal relationships as likely or certain to be harmed if they comply with TDF laws (thus keeping them away from home), yet a crash or sanctions are unlikely to occur if they breach TDF laws (enabling them to get home earlier), then the driver’s attitude and intentions regarding TDF are likely to be influenced by the more certain outcome. As participants reported, driver behaviour is likely to be consistent with that which avoids the certainty of harming personal relationships. This suggests that they are more likely to form non-compliant intentions that enable them to return home earlier.

### 4.1. Family-Friendly Work Practices and Safety Culture

The predictive capacity of the TPB regarding compliance intentions in this context highlights the importance of more flexible, family-friendly work schedules. Respondents indicated that such scheduling would allow more time at home to nurture personal or family relationships and reduce the risks of family breakdown. According to Arnold and Hartley [70] and McCartt et al. [71], arduous work schedules increase the likelihood that drivers will breach fatigue laws, thereby leading to fatigued driving. When work schedules are performed back-to-back, it is likely that this will increase TDF risks because of the additional pressure this places on drivers to “*get the job done*” and to return home [12,27,72] (p. 32). Tucker and Folkard [60] and Arnold et al. [73] suggest that giving drivers greater control over work schedules will address this problem. While such initiatives have clear merit, they are yet to be tested within the heavy vehicle sector. Managers spoke of their efforts to support drivers facing personal problems that affect fatigue management, yet this was contingent upon drivers disclosing fatigue-related issues. This, however, is an action that drivers are reluctant to do [6]. While it is unlikely managers could cover all contingencies when personal or family issues arise, they nonetheless have a duty of care to drivers to proactively manage fatigue and the risks it poses for employee and public health and safety. This is recognised within the Heavy Vehicle National Law [14] (Section 26C).

There is evidence which suggests that when a positive safety culture exists within an organisation regarding communication and rest compliance, employees may be more inclined to disclose issues such as those related to fatigue risks [67,74]. For example, a driver who experiences sleep loss because of a personal issue may be more inclined to disclose the issue to their manager. This may be more likely if they work in a positive safety environment where disclosure is likely to result in them receiving support. This may include drivers having access to alternate transport home without a loss of income, which negates the need for drivers to engage in risky TDF behaviours. Honn et al. [54], however, acknowledge that creating a positive safety culture via good fatigue risk management systems is resource intensive, and often beyond the capacity of smaller operators.

According to Arboleda et al. [75], Naevestad, Blom and Philips [76] and Mouton, Goedhals-Gerber and Bod, [77], when management recognises safety, and commits to it in terms of practice, it is the first step towards the development of a safety culture. In addition, Mouton et al. [77] suggest that support for a safety culture within the freight industry may lead to greater dedication and pride amongst truck drivers in their work, and accordingly, better compliance with rules and regulations regarding risk. This is important because Naevestad et al. [76] and Washburn, Kueny and Murray [78] suggest that a relationship exists between safety culture and accident risk. They recommend the adoption of several management practices that can increase safety culture, such as developing company safety policies, implementing well-developed training programs to increase driver competence and knowledge. and encouraging drivers to stop if they perceive it is unsafe to continue [76]. These practices, however, were discussed by several participants as being difficult to resolve because the Australian transport industry is dominated by small operators, with 70–80% of transport companies operating with only one or two trucks [79,80], who lack the resources of larger companies. While better scheduling may reduce occupational risks (because drivers will have greater autonomy to respond to personal issues affecting TDF compliance) [26], such scheduling can be *“difficult to redesign”* [81] (p. 185) and may only be an option available to larger companies.

Although this may resolve some issues relating to TDF compliance, the impact of external influences on drivers’ compliance or non-compliance with TDF regulations cannot be ignored. For example, when drivers experience financial hardship due to delays in loading and unloading freight, it is likely to increase TDF risk-related behaviour. This is because under the TPB, external influences (such as loading delays) become behavioural controls that are used to determine the performance of a given behaviour. In this instance, intention to rest and comply with TDF regulation and not engage in risk-related behaviours becomes shaped by factors over which drivers may not have complete volitional control. Yet, these factors may be perceived as being easy (compliance when there is no delay) or difficult (compliance when there is a delay) to perform when determining an outcome.

If it is easier for a driver to skip rest breaks, exceed driving hours and manipulate work diary records to maintain work schedules and sustain income, then it is likely that they will determine which behavioural controls facilitate or impede the action. In this instance, it is to complete the work and get paid. To overcome this issue, drivers could be paid for the time they spend waiting for freight to be loaded or unloaded. Such a suggestion has merit because it could speed up loading and unloading and transfer this economic risk from drivers back to the transport customer [82,83]. Ultimately, it could give drivers greater control over their income and assist implementation of TDF management and compliance risks.

While this type of organisational change is likely to reduce TDF risk-related behaviour, the findings from this study indicate that the laws, in their current form, are perceived as being inflexible and incompatible with human experiences of fatigue. This can result in perverse outcomes whereby drivers are ”resting” because of regulatory requirements and not because they are fatigued. It may result in drivers not taking needed breaks because they are not ”scheduled”. Of particular concern are financial sanctions for minor clerical breaches, because these are perceived as exacerbating TDF risk-related behaviour as drivers address or dispute legal matters. Legislators should also consider changes to TDF laws to redefine the term “rest” to ensure that recuperative rest is more likely to occur. As part of such changes, the standard rest period could be increased to 20 min to enable a 15 min power nap and a 5 min establish/resume period. For example, current sleep science indicates that brief naps of this duration are optimal and support immediate increases in attention and cognitive function [60,84,85].

The study findings highlight that change must occur within enforcement organisations to actively hold to account other parties in the ”chain of responsibility” [14] (Section 5). Driver-focused enforcement enables transport managers and companies to engage in a form of ”risk transfer” [82,83] which transfers occupational risk (crashes and sanctions) from themselves and their company to the drivers. Arnold and Hartley [70] (p. 3) argue that truck drivers are *“the unfortunate inheritors of poor decision making on the part of their companies”* The continuance of this approach provides little incentive for companies and the industry to engage in the kind of sector-wide reforms to operational work environments that are needed to support drivers’ work health and safety and that of the travelling public. Several researchers have argued that the operational environment in which truck drivers work plays a key role in TDF behaviour and that leaves many drivers fatigued and at an increased risk of a TDF-related crash [86,87,88,89]. When the work environment is shaped around TDF, and rest and recovery, it is likely that this will positively affect occupational wellbeing and workplace safety culture more broadly. For example, Crum et al. [86] suggest that modifying the work environment to provide drivers with adequate recovery time between shifts and putting drivers on regular time schedules is likely to reduce fatigue. Furthermore, they suggest that drivers should use the additional recovery time to obtain adequate rest, and that for this to be successful, transport companies and transport customers should work together to improve scheduling and the performance of loading and unloading activities [86].

### 4.2. Technology Promising Better Fatigue Management

Participants highlighted that better organisational TDF practices and technologies often fostered more positive attitudes towards TDF management. These included flexible scheduling and upgrading trucks with comfortable bedding to enable restful sleep. Company managed e-diaries and e-monitors may also give drivers greater control over work and rest. Some drivers reported that it can empower them to resist pressure from managers who may, intentionally or inadvertently, ask drivers to engage in non-compliant activity. In fact, there were some indications that this technology was changing the working relationship of drivers and managers regarding TDF. This is because both need to operate according to this objective, and do so with a real-time data source as their common reference. However, access to these devices may be challenging for drivers employed in smaller companies [54]. If companies implement electronic monitoring devices that enable drivers to better manage TDF and compliance with TDF laws, then it is likely that drivers will possess positive attitudes towards such equipment. This will likely reduce TDF risk-related behaviour.

Under the TPB, a positive disposition towards such practices is supported by the concept of perceived behavioural controls. This is because as a behavioural control, e-monitors and e-diaries facilitate the ease or difficulty of compliant and non-compliant behaviour. This shifts the locus of control of intention towards the driver. For example, when a driver’s TDF-related behaviour is facilitated by flexible scheduling (greater personal control over TDF management), e-diaries (greater control over TDF compliance) and trucks equipped with comfortable bedding (better rest options), the driver is likely to perceive greater ease when enacting TDF-compliant behaviours.

A greater level of control over rest-related factors may enable the driver to form a positive intention to not engage in TDF risk-related behaviour. While the use of e-monitors and e-diaries may reduce TDF risk, it is important to note that electronic monitoring by enforcement organisations, such as via the National Safety Camera Network, are not viewed as being helpful for drivers. This type of monitoring is perceived as being used to enforce a prescriptive system of fatigue compliance that lacks flexibility. The physiological and work-related challenges truck drivers identified in this instance do not help them counter situations that may affect compliance [90].

The findings from this study suggest that personal challenges influence truck drivers’ and transport managers’ intentions regarding compliance with fatigue regulation. The findings determine that these challenges intrinsically affect how they manage health and safety risks relating to fatigue. This adds new knowledge to the limited Australian and international literature regarding TDF behaviour and management. This study also identified various areas where further research is required. To capture potential personal and family circumstances affecting TDF behaviour more broadly, future research could increase the sample of women truck drivers, families of drivers, and drivers and managers identified as being a member of a diverse group or from culturally and linguistically diverse backgrounds. In addition, as new technology emerges, studies focusing on how truck drivers and transport managers perceive technology could identify new information regarding how this influences intentions and behaviours regarding TDF compliance and management. This could include in situ, real-time monitoring and data collection, along with data outputs and drivers’ perceptions of introduced driver-support technologies. These inclusions would enable a higher level of confidence in the generalisability of the findings.

### 4.3. Recommendations

Based on the findings, the following seven recommendations are actionable items that may promote truck drivers’ health and safety and reduce TDF-related public health risks. The findings in this study support three recommendations found in other studies, (recommendations 3/4/6), and four unique recommendations emerge from this research. For example, Recommendation #1 identifies a need for strategies that address personal factors affecting TDF-related safety and the health of truck drivers. Recommendation #2 also identifies specific personal factors (relating to the families of truck drivers) that, when addressed, can better support them to manage fatigue and health-related risks. Recommendation #5 proposes that legislators must redefine ”rest” within legal parameters—this is an issue that has not been previously identified. Recommendation #7 includes the need for the industry and government to recognise the importance of (and support the uptake of) new electronic monitoring technology that will assist truck drivers, including owner-drivers or small operators, to better manage fatigue. As such, it is recommended that industry peak bodies, company managers, transport unions and truck drivers perform the following:

(1) Co-develop strategies within fatigue management systems to account for personal factors likely to influence drivers’ intentions regarding TDF and support them to engage in safer and healthier TDF practices.
(2) Facilitate the development, provision and promotion of standardized education resources and programs for truck drivers’ families so they can better support drivers to maintain restorative rest practices and healthy lifestyles.
(3) Implement flexible scheduling to better enable truck drivers to rest as required.
(4) Furthermore, it is recommended that industry peak bodies and the government perform the following: Reduce or prohibit the use of incentive payments in favour of hourly payments that include remuneration for delays and waiting times when loading or unloading.
(5) Redefine the regulatory definition of ”rest” to ensure it directs restorative rest during both pre-shift and in-shift rest periods. This could include allowing rest periods at any time after 2 h of driving, and 20 min rest periods to enable a 15 min ”power nap”. For such provisions to be workable, they may need to occur in concert with the infrastructure improvements (e.g., dedicated truck driver rest areas) identified in recent research [6].
(6) Encourage compliance organisations to refocus activities towards others in the chain of responsibility. This can incentivise companies to engage in safer TDF practices that support drivers and reduce the inequity of driver-focused enforcement.
(7) Play a leading role in financing, developing and promoting industry-standardised training and technologies such as e-diaries and e-monitors for all drivers and companies, with a particular focus on enabling small companies to access these resources.

### 4.4. Limitations and Strengths

All research is subject to socially desirable responses, and the possibility that self-reported data can lead to inaccurate results. As such, it is important to acknowledge that all the participants (regardless of having been recruited by their respective organisation or from listening to a podcast) may have self-selected responses to the interview questions based on their interest in this topic. This may have generated bias in their responses. As stated, however, it was unknown how participants in the final sample were informed about the research, and whilst this may have skewed the findings, the direction of bias that may have informed the findings was unable to be quantified. Their views, therefore, may not reflect the wider perceptions of professional cohorts across the transport industry. It is also acknowledged that the findings may be subject to potential bias because of the non-random selection methods used by the organisations in the selection of long-haul drivers and managers, and due to the self-interest in this study of those participants who responded to the primary researcher after listening to the podcasts. As such, the findings may not be generalisable.

In addition, the small number of participants in this study may have limited the findings. Future research that replicates this study, therefore, can overcome these limitations by increasing the sample size to represent a national sample, and utilising additional methodologies such as a participant observation study of truck drivers and transport managers in situ, as well as an online survey using grouped-answer responses to mask sensitive questions among others. This multimethod approach and the use of a deliberate integration of qualitative and quantitative approaches within the same study will strengthen the findings and address socially desirable responses. Being better informed about how participants acquire information about involvement in research will also provide an opportunity to identify the direction and magnitude of bias between participants recruited by different means. This will increase the likelihood that the findings will be generalisable and representative of truck drivers and transport managers across Australia and internationally. Despite these limitations, the current findings provide important primary data regarding Australian truck drivers’ and transport managers’ perceptions of how personal and contextual challenges are likely to influence their intentions regarding compliance with fatigue regulations.

## 5. Conclusions

By using semi-structured interviews and applying the TPB to explore how personal behavioural determinants may influence truck drivers’ responses to TDF, the findings from this study present new knowledge to the truck driver fatigue literature. For example, the findings suggest that personal factors such as familial influences and personal financial viability are likely to be core elements that influence TDF compliance behaviour. This includes how they manage health-related risks and the effect this may have on drivers and the public. Personal circumstances, therefore, are identified as a predictive factor in determining TDF-related behaviour. The findings also suggest that contextual factors such as inflexible delivery schedules are also likely to shape drivers’ TDF behaviour. Building greater flexibility into TDF regulations and delivery schedules is likely to reduce time pressures and financial concerns for drivers because it gives them greater control in response to TDF cues. Similarly, the use of incentive payments needs careful reconsideration, because this form of remuneration is associated with higher TDF crash risks. Assisting transport companies (regardless of size) to implement standardised industry-wide TDF training and to adopt technologies such as e-diaries and e-monitors is likely to benefit companies and drivers by reducing TDF risk-related behaviours. It may also transform the chain of responsibility regarding TDF compliance. By broadening the obligation of TDF best practice to all key stakeholders, it will foster more positive attitudes towards TDF compliance and regulation, thereby reducing the likelihood of risk-related road crashes.

### Practical and Theoretical Contributions

The findings in this study contribute practical solutions regarding how transport companies and managers can support truck drivers in relation to TDF risk reduction. This includes fostering open communication about the personal and contextual risk factors that are likely to increase TDF, providing flexibility in terms of work schedules and promoting a supportive culture of safety regarding TDF that begins with TDF recognition, increased education and training, TDF wellness programs, and peer support systems. Providing necessary resources such as TDF-related technologies can potentially increase the likelihood that truck drivers are able to drive safely and perform delivery tasks that are monitored and managed by all parties in the chain of responsibility.

The findings in this study also provide theoretical contributions regarding how the TPB can aid in identifying personal and contextual factors that are likely to affect TDF. For example, subjective norms in relation to the influence of key people in a driver’s life are likely to strongly influence TDF-related risk behaviour. Whilst perceived behavioural controls regarding how easy or difficult it is for a driver to comply with TDF regulations certainly influence the intention of drivers to follow TDF-related rules, it is the influence of personal rather than contextual factors that are identified as being the strongest component of the TPB that intrinsically affect intention (in this instance, intention to comply with TDF regulations). In this study, the relationship between TDF risk-related behaviour and subjective norms connects this component of the theory to practice because of the challenges truck drivers face when weighing up TDF risks and demands placed upon them as personal factors arise. The identification of subjective norms as being a key influence on intention to behave (in this instance, intention to comply with TDF regulations) advances the understanding of subjective norms in relation to intention and their relationship to the theory, thereby improving the understanding of intention to behave in the context of truck driving and TDF risk.

## Figures and Tables

**Table 1:** Participant characteristics (N = 44).

	Male	Female
Role:		
Truck Drivers	30	6
Transport Managers	8	
Average Years of Service:		
Truck Drivers	26	12
Transport Managers	16	
Average Age:		
Truck Drivers (24–76 yrs)	56	54
Transport Managers (30–77 yrs)	55	

**Table 2:** Geographical locations of truck drivers (N = 36).

Geographical Location	Total
New South Wales	7
Queensland	14
Victoria	5
South Australia	0
Australian Capital Territory	0
Tasmania	0
Western Australia	9
Northern Territory	1

**Table 3:** Jurisdiction type (N = 36).

Jurisdiction Type	Total Number of Drivers
Heavy Vehicle National Law	26
Workplace Health and Safety	10

**Table 4:** Payment methods (N = 36).

		Total Number of Drivers	%
**Payment Category**	Per trip	9	25
	Per km	14	39
	Per tonne	1	3
	Per day	1	3
	Hourly	11	30
**Payment Motive**	Incentive	24	67
	Non-incentive	12	33

**Table 5:** Load types (N = 36).

Load Types:	Total Number of Drivers	%
Bulk dangerous goods	4	11
Refrigerated goods	5	14
General freight	17	47
Bulk loads (soil, grain etc)	5	14
Oversize/Over-mass loads	4	11
Livestock	1	3

## Data Availability

The raw data supporting the conclusions of this article will be made available by the authors on request.

## Abbreviations

The following abbreviations are used in this manuscript:

GVM	Gross Vehicle Mass
TDF	Truck Driver Fatigue
TPB	Theory of Planned Behaviour
HVNL	Heavy Vehicle National Law
WHS	Workplace Health and Safety

## Appendix A. Participation Texts—Truck Drivers and Transport Managers

Dear Sir/Madam,

I am a PhD student at Western Sydney University, and I am researching truck driver fatigue. The aim of my research is to understand truck driver’s beliefs and attitudes towards driver fatigue from the driver’s perspective, when driving long haul trucks. This research project has ethics approval from the Western Sydney University Ethics Committee, approval number: H15319.

As such, I am seeking truck drivers who work in long haul operations to participate in a short interview.

If you are interested in participating in an interview, please see the attached information sheet which contains information detailing the research and how to contact me.

I look forward to hearing from you. Thank you for considering being part of my research.

Dear Sir/Madam,

I am a PhD student at Western Sydney University, and I am researching truck driver fatigue. The aim of my research is to understand the beliefs and attitudes of truck drivers towards driver fatigue as well as the perspective of their controllers/managers who are responsible for managing truck driver fatigue. This research project has ethics approval from the Western Sydney University Ethics Committee, approval number: H15319.

As such, I am seeking Controllers or Managers of truck drivers who work in long haul operations to participate in a short interview.

If you are interested in participating in an interview, please see the attached information sheet which contains information detailing the research and how to contact me.

I look forward to hearing from you. Thank you for considering being part of my research.

## Appendix B. Interview Schedule—Truck Drivers

*(Interviewer instructions/reminder) Each interview will last between 30 to 60 min depending on the elaboration of the responses given. Spontaneous or prompt questions not listed in this schedule may be asked to encourage narrative and to ensure responses have been fully understood by the interviewer. The interview questions ask separate questions in respect of Heavy Vehicle National Law (HVNL) jurisdictions and Workplace Health and Safety (WHS) jurisdictions as identified throughout. Italicised questions are to guide to the interviewer.*

**Thank you for your time today. The purpose of this interview is to gather information regarding your beliefs and attitudes towards truck driver fatigue and its management. Just a reminder, that everything you say is completely confidential and you will not be identified in any way. You are free to withdraw from this interview at any time.**

**Do you give your consent to participate in this study? Y/N**

**Do you give your consent for me to proceed with the recording? Y/N**

1. Demographics and Experience

1.1. How old are you?

1.2. Do you identify as Male/Female/Other?

1.3. Were you born in Australia? *(What country were you born in? What is your cultural heritage? Is English your first language? Do you identify as indigenous Aboriginal or TSI?)*

1.4. What is your highest level of education?

1.5. Do you live in the same state as your driver base? Y/N

1.6. How long have you been working as a long-haul truck driver? (total years)

1.7. Have you had any breaks during your career where you worked in a job other than truck driving? If so, when were these breaks and how long did you spend away from truck driving?

1.8. What kinds of transport companies have you worked for? (small operators/major companies, etc.)

1.9. Are you hauling general freight/refrigerated produce/raw materials/other?

1.10. Are you currently an employed driver or self-employed?

*(To identify different experiences between employed drivers and self-employed owner/drivers)*

1.11. Are you currently paid per hour, per trip, per km, *(or main method)* etc.?

1.12. Standard, BFM, AFM?

2. Learning about truck driver fatigue and fatigue management

2.1. Are you aware of the physical effects of truck driver fatigue? Do you think most truck drivers can describe them? *(To identify the extent of truck drivers existing knowledge about the physical effects of truck driver fatigue.)*

***If the participant answers ‘Yes’ continue from Q2.2 to Q2.4 only. If the participant answers ‘No’, skip to Q 2.5.***

2.2. How do you think most truck drivers learn about the **physical effects of fatigue on truck drivers?** *For example, training courses, internet, friends, other truck drivers, managers, trade unions or associations, self-taught/research, other sources? (To identify where they sourced knowledge about the physical effect of truck driver fatigue and to assess the breadth of their information sources.)*

2.3. How do you think most truck drivers learn about **the laws regarding truck driver fatigue management?** *For example, training courses, internet, friends, other truck drivers, managers, trade unions or associations, self-taught/research, other sources? (To identify where they sourced their knowledge about the laws regarding truck driver fatigue laws and to assess the breadth of their information sources.)*

2.4. How do you think most truck drivers learn about **company rules and operational guidelines regarding truck driver fatigue management?** *For example, training courses, internet, friends, other truck drivers, managers, trade unions or associations, self-taught/research, other sources? (To identify where they sourced their knowledge about company rules regarding truck driver fatigue and to assess the breadth of their information sources.)*

***If the participant has answered ‘No’ to Q 2.1 add the following preamble relevant to the participant’s jurisdiction:***

***(In HVNL jurisdictions,*** Heavy Vehicle National Law describes fatigue as ***”including but not being limited to feeling sleepy, feeling physically or mentally tired, weary, or drowsy and feeling exhausted or lacking energy and behaving in a way consistent with any or all these experiences”.)***

(***For WHS jurisdictions***, Safework Australia defines fatigue as ***”…. a state of mental and/or physical exhaustion that reduces a person’s ability to perform work safely and effectively”.)***

2.5. Having described the definition of fatigue, do you think this is something most truck drivers are aware of? *(To identify their perception of truck divers’ understanding of fatigue.)*

**Coding Schema—Interview Items and TPB Components**

3. Beliefs, attitude—RQ 2—How these shape compliance/non-compliance

*Belief: ”Something that is accepted, considered to be true” (Merriam-Webster, 2022)* [91].

*Attitude: ”a disposition to respond favourably or unfavourably to an object, person, institution or event’ that derives ‘reasonably from accessible beliefs about the behaviour”. Beliefs and attitudes are subject to change (Ajzen, p3, p30, 137)* [36].

***(If participants answered, ‘Yes’ to Q 2.1, ask the series of questions marked with ‘a’. If participants answered ‘No’ to Q 2.1, ask the series of questions marked with ‘b’.)***

**_ _ _**

3.1.a. What do you think/believe are the main signs of driver fatigue? *(To identify their understanding of the definition of fatigue relevant to research and legislative definitions.)*

3.2.a. How do you think/believe most truck drivers experience it? What is usually happening when they experience fatigue? *For example, feeling sleepy, struggling to stay awake, yawning, can’t concentrate, feeling exhausted or lacking energy, etc. (This is for me to compare their understanding of driver fatigue in relation to the formalised definition/s of fatigue for later use in my discussion chapter.)*

3.3.a. In your opinion, is driver fatigue an important issue for truck drivers? *(To ascertain their belief about the importance of truck driver fatigue as an issue for them and their cohort.)*

3.4.a. In your opinion, do **the physical effects of truck driver fatigue** affect their ability to do the job properly? *(To identify their beliefs about the significance of the physical effects of fatigue on truck drivers.)*

3.5.a. Could you give me an example of how you think/believe driver fatigue could increase risks for truck drivers? *(To ascertain their capacity to identify the risks associated with truck driver fatigue.)*

3.6.a. In your opinion, what are most truck drivers’ attitudes towards **the truck driver fatigue laws**? *For example, are they effective in helping truck drivers manage their fatigue? Are they easy or difficult to comply with? Do they help truck drivers get the job done? (To ascertain their attitudes towards truck driver fatigue laws, perceptions of effectiveness and how these laws affect their work.)*

3.7.a. In your opinion, what are most truck drivers’ attitudes towards **company rules** about driver fatigue? *For example, do they think they are effective in helping truck drivers manage their fatigue? Do they think they are easy or difficult to comply with? Do they help truck drivers get the job done? (To ascertain their attitudes towards company rules regarding truck driver fatigue, perceptions of effectiveness and how these rules affect their work.)*

3.8.a. In your opinion, how important is it for truck drivers to comply with truck driver fatigue laws? *(To assess their own beliefs about the importance of compliance with truck driver fatigue laws.)*

3.9.a. In your opinion, how important is it for truck drivers to comply with company rules regarding driver fatigue? *(To assess their own beliefs about the importance of compliance with company rules regarding driver fatigue.)*

3.10.a. Thinking about truck driver fatigue and the laws and company rules about it, what information source do you trust the most? Why? *(Relates to questions 2.2, 2.3 and 2.4; to assess where they sourced their most trusted information about truck driver fatigue.)*

3.11.a. In your opinion, do most companies manage truck driver fatigue in an effective way? What main strategies (if any) do they use to manage driver fatigue? *For example, do they offer training programs? Do they distribute truck driver fatigue-related advertising material, inter-company emails,* etc.*? Is fatigue mentioned during induction programs? Are most truck drivers encouraged to follow the instructions in their work diary? (In WHS jurisdictions, do truck drivers receive any training or induction information? Are they informed about driver fatigue management plans,* etc.*)?* In your opinion, are these strategies effective? Why/why not? *(To assess beliefs about how truck driver fatigue is managed.)*

3.12.a. Is there one strategy that stands out as being really helpful to you? *For example, what do you think the company does best?* Is there anything you think/believe could be done better?

*For example, is there one thing that stands out as being unhelpful or a waste of time*? *(To assess beliefs about how truck driver fatigue is managed.)*

3.13.a. Having now discussed driver fatigue, in your opinion, do most truck drivers think/believe truck driver fatigue is a priority/safety issue? *For example, on a scale of 1 to 10 where 1 is the least important and 10 is the most important, how would they rate truck driver fatigue as a priority issue?* Why? *(To assess beliefs about how truck drivers perceive the importance of truck driver fatigue.)*

3.14.a. Having now discussed driver fatigue in relation to rules and regulations, what do you see as the main things that contribute to truck driver fatigue? *(To assess their own belief about causes of truck driver fatigue.)*

3.15.a. Are there ways to manage it better than it is managed now? If yes, how? *(To assess their own belief about solutions regarding truck driver fatigue.)*

**_ _ _ _**

***Skip alternative to Q 3.1a as it is not relevant to participants who answer ‘No’ to Q 2.1.***

***Skip alternative to Q 3.2a as it is not relevant to participants who answer ‘No’ to Q 2.1.***

3.3.b. Having defined fatigue, do think/believe it is an important issue for truck drivers? *(To ascertain their belief about the importance of truck driver fatigue as an issue for them and their cohort.)*

3.4.b. Having defined fatigue, do you think/believe **the physical effects of truck driver fatigue** could affect truck drivers’ ability to do the job properly? *(To identify their beliefs about the significance of the physical effects of truck driver fatigue.)*

3.5.b. Could you give me an example of how you think/believe driver fatigue could increase risks for truck drivers? *(To ascertain their capacity to identify risks associated with truck driver fatigue.)*

3.6.b. Having defined fatigue, how do you think/believe **truck driver fatigue laws** might affect truck drivers? *For example, could they help truck drivers manage their fatigue? Are they easy or difficult to comply with? Do they help truck drivers get the job done? (To ascertain their attitudes towards truck driver fatigue laws, perceptions of effectiveness and how the laws affect their work.)*

3.7.b. Having defined fatigue, how do you think/believe **company rules** might affect truck drivers? *For example, could they be effective in helping truck drivers manage their fatigue? Are they easy or difficult to comply with? Could they help truck drivers get the job done? (To ascertain their attitudes towards company rules regarding truck driver fatigue, perceptions of effectiveness and how these rules may affect their work.)*

3.8.b. Having defined fatigue, in your opinion, how important is it for truck drivers to comply with **truck driver fatigue laws**? *(To assess their own beliefs about the importance of compliance with truck driver fatigue laws.)*

3.9.b. Having defined fatigue, in your opinion, how important is it for truck drivers to comply with **company rules regarding truck driver fatigue**? *(To assess their own beliefs about the importance of compliance with company rules about truck driver fatigue.)*

***Skip alternative to Q 3.10a as it is not relevant to participants who answer ‘No’ to Q 2.1.***

3.11.b. If other truck drivers are unaware of driver fatigue, why do you think/believe they are not aware? *For example, do other companies not offer training programs? Do they not distribute truck driver fatigue-related advertising material, inter-company emails,* etc.*? Is fatigue not mentioned during induction programs? Do other truck drivers not notice the instructions in their work diary? (In WHS jurisdictions, do truck drivers not receive any training or induction information? Are they not informed about driver fatigue management plans,* etc.*)?* What do you think/believe could be done better? *(To assess beliefs about how truck driver fatigue is managed.)*

***Skip alternative to Q 3.12a as it is not relevant to participants who answer ‘No’ to Q 2.1.***

***Skip alternative to Q 3.13a as it is not relevant to participants who answer ‘No’ to Q 2.1.***

3.14.b. Having now discussed driver fatigue in relation to rules and regulations, what do you see as the main things that contribute to truck driver fatigue? *(To assess their own belief about causes of truck driver fatigue.)*

3.15.b. Having defined fatigue, do you think there are ways to manage it better than it is managed now? If yes, how? *(To assess their own belief about solutions regarding truck driver fatigue.)*

4. Subjective norms—RQ 3—Key Influences on compliance/non-compliance

*(In accordance with the Theory of Planned Behaviour, for certain cohorts and professions, there may be a fine line between subjective norms and perceived behavioural controls—for law enforcement and policing practices this is important to consider and needs careful unpacking in the interviews and related questions.)*

***(If participants answered, ‘Yes’ to Q 2.1, ask the series of questions marked with ‘a’. If participants answered ‘No’ to Q 2.1, ask the series of questions marked with ‘b’.)***

4.1.a. Is truck driver fatigue something that is discussed in truck drivers’ workplaces or amongst truck drivers themselves? How often is it discussed? What triggers these discussions? *For example, a crash, a fine being issued,* etc.*? (To ascertain potential origins and recency of subjective norms regarding truck driver fatigue.)*

4.2.a. In your opinion, what are the strongest influences on truck drivers’ perceptions of **truck driver fatigue?** What shapes their ideas the most? *For example, could it be the opinion of other drivers, the manager or personal experience—some drivers’ perceptions might involve experiences where they felt fatigued at work, while others might report experiences of long periods of driving without feeling fatigued? How do you think/believe this might influence their perceptions of the physical effects of fatigue? (To identify the influences on the co-worker cohort about the physical effects of truck driver fatigue.)*

4.3.a. In your opinion, what are the strongest influences on the way truck drivers think about **compliance with truck driver fatigue management requirements**? *For example, are truck drivers more influenced by company rules or fatigue management laws or are these equally influential? Why/why not? Do the shared opinions of truck drivers influence each other’s responses to truck driver fatigue? (To identify the influences within truck drivers’ workplaces regarding truck driver fatigue laws and compliance.)*

4.4.a. Do you think/believe truck drivers are influenced about compliance issues by other factors relating to driver fatigue, such as things that may heighten their awareness of it? *For example, their awareness of road crashes, injury, death,* etc., *due to driver fatigue?* Do you think/believe these types of factors influence their compliance or non-compliance? Are there any other things or influences that may affect truck drivers’ compliance or non-compliance with driver fatigue rules and regulations, such as use of legal or illegal drugs/medication regarding fatigue? *(To identify their understanding of other influences on truck drivers and truck driver fatigue.)*

4.5.a. Whilst truck drivers are influenced by their controllers and the operational guidelines of the company in terms of workplace rules and practices, who or what do you think/believe would be other influences that may affect truck drivers’ compliance or non-compliance with driver fatigue laws and company rules? *For example, friends, family, partners, financial concerns, loads with short delivery timeframes, etc? (To identify the influence of significant others, for example, friends, family, partners, financial concerns,* etc.*, on a truck driver’s compliance/non-compliance and attitudes/beliefs regarding truck driver fatigue laws and company rules.)* What loads have the shortest delivery timeframes?

**_ _ _ _**

***Skip alternative to Q 4.1a as it is not relevant to participants who answer ‘No’ to Q 2.1.***

4.2.b. Having defined fatigue, in your opinion, what do you think/believe might be the strongest influence how other truck drivers’ might perceive **truck driver fatigue?** *For example, could it be the opinion of other drivers, the manager, or personal experience—some drivers’ perceptions might involve experiences where they felt fatigued at work, while others might report experiences of long periods of driving without feeling fatigued? How do you think/believe this might influence their perceptions of the physical effects of fatigue? (To identify the influences on the co-worker cohort about the physical effects of truck driver fatigue.)*

4.3.b. Having defined fatigue, in your opinion, what might be the strongest influences on the way truck drivers think about **compliance with truck driver fatigue management requirements**? *For example, are truck drivers more influenced by company rules or fatigue management laws or are these equally influential? Why/why not? Do the shared opinions of truck drivers influence each other’s responses to truck driver fatigue? (To identify the influences within truck drivers’ workplaces regarding truck driver fatigue laws and compliance.)*

4.4.b. Having defined driver fatigue, do you think/believe truck drivers could be influenced by other factors relating to driver fatigue, such as things that may heighten their awareness of it, to increase compliance? *For example, their awareness of road crashes, injury, death,* etc., *due to driver fatigue?* Do you think/believe these types of factors could influence their compliance or non-compliance? Are there any other things or influences that may affect truck drivers’ compliance or non-compliance with driver fatigue rules and regulations, such as use of legal or illegal drugs/medication regarding fatigue*? (To identify their understanding of other influences on truck drivers and truck driver fatigue.)*

4.5.b. Whilst truck drivers are influenced by their controllers and the operational guidelines of the company in terms of workplace rules and practices, having now defined driver fatigue, who or what do you think/believe would be other influences that may affect truck drivers’ compliance or non-compliance with driver fatigue laws and company rules? *For example, friends, family, partners, financial concerns,* etc.*?(To identify the influence of significant others, for example, friends, family, partners, financial concerns,* etc.*, on a truck driver’s compliance/non-compliance and attitudes/beliefs regarding truck driver fatigue laws and company rules.)*

5. Perceptions of behavioural control—RQ 4—How easy/difficult it is to comply/not comply with the behaviour (compliance/non-compliance with driver fatigue guidelines/laws/company rules)

***All questions apply regardless of the participants answer to Q 2.1.***

5.1. Having discussed and identified truck driver fatigue, in your opinion, when truck drivers start their shift do you think/believe most of them intend to comply with **truck driver fatigue laws**? *(To ascertain initial short-term intentions regarding compliance or non-compliance with truck driver fatigue laws.)*

5.2. Having discussed and identified truck driver fatigue, in your opinion, when truck drivers start their shift do you think/believe most of them intend to comply with **company rules** about truck driver fatigue? *(To ascertain initial short-term intentions regarding company rules about truck driver fatigue.)*

5.3. In your opinion, what kind of things might change a truck driver’s intentions about complying with truck driver fatigue laws and/or company rules during a shift? *For example, during a shift if there is a delay, or if the driver has difficulty finding a suitable rest area, how might that influence their intentions to comply with truck driver fatigue laws and/or company rules?* Can you give me some examples of the types of barriers that that you think may disrupt drivers’ intentions and behaviours regarding compliance with fatigue laws and company rules? What is it that can make compliance easy or difficult? *(To ascertain triggers that may affect the perception of behavioural control, intentions and behaviour of truck drivers regarding truck driver fatigue compliance.)*

5.4. When truck drivers are on the road, how easy or difficult do you think/believe it would be for them to ignore rest requirements? *For example, would it be easy or difficult for them to ignore the requirements of laws and/or company rules regarding truck driver fatigue? Could they ignore instructions in their work diary? Could they ignore advisory road signs? Could they ignore instructions from their manager/controller? (To identify truck drivers’ perceptions of behavioural control regarding compliance or non-compliance whilst on the road.)*

5.5. When finishing their shift, if drivers report to managers/controllers, in your opinion, how easy or difficult would it be for truck drivers to hide their compliance or non-compliance with driver fatigue laws and company rules? If managers/controllers are aware that drivers have not complied with driver fatigue laws and company rules, in your opinion, how easy or difficult might it be for them to overlook this? *(To ascertain how easy or difficult it is in reality, for drivers and controllers, to ignore the issue of compliance/non-compliance with laws and company rules regarding truck driver fatigue.)*

5.6. At the start of their career, how easy or difficult do you think/believe it would be for truck drivers to ignore rules and regulations? Would compliance become easier or more difficult over time? *For example, does it become easier or more difficult to follow laws and company rules starting out, or mid-career or towards the end of their career? Is it easier or more difficult for truck drivers with more or less experience to comply with/follow/uphold driver fatigue laws and company rules? (To ascertain ease or difficulty of compliance with and management of truck driver fatigue laws and company rules at different stages of their careers.)*

5.7. Having discussed how easy or difficult compliance or non-compliance would be, what do you think/believe would be the key thing that would shape drivers’ intention to comply or not comply? *For example, increased knowledge and experience? Working as an employed driver or an owner/driver? (To identify temporal and/or other factors that may affect the ease or difficulty of compliance/non-compliance as well as to identify key influences on intention.)*

5.8. Having discussed and identified truck driver fatigue, in your opinion, when thinking about compliance/non-compliance and driver fatigue management, do most truck drivers think about law enforcement of this issue, and do you think enforcement influences their intention to comply or not comply with driver fatigue laws? *For example, is awareness of law enforcement involvement in this issue likely to make truck drivers think about taking rest breaks? (To ascertain the perceived behavioural controls that may encourage a truck driver to think more about truck driver fatigue and compliance or non-compliance with truck driver fatigue laws—this again is about how intention is shaped.)* Do you see law enforcement activity when you are out on the road? Police/NHVR/WHS? How does it make you feel when you see it?

5.9. In your opinion, how easy or difficult do current law enforcement practices make it for truck drivers to comply with the laws regarding truck driver fatigue? Why? What do you think/believe enforcement might mean for truck drivers in terms of the law if they do not comply? *For example, fines, arrest, other outcomes? (To ascertain, in the context of perceptions of enforcement practices, the extent of control these perceptions have on truck drivers’ intentions and behaviour regarding compliance or non-compliance with truck driver fatigue laws and company rules, and whether the awareness of law enforcement shapes their workplace practices.)*

5.10. In your opinion, what do you think/believe would be the main thing that shapes the overall intentions of truck drivers regarding compliance with driver fatigue laws and company rules etc.? *For example, a desire to comply with the law, a desire to comply with company rules, safety concerns, concerns of family or friends, financial concerns, instructions from their manager/controller,* etc.*? (To ascertain the most significant influence that shapes the intention of truck drivers to uphold laws and company rules regarding truck driver fatigue.)*

**Thank you for your time today in participating in this interview. Please be assured that all the data you have supplied will be de-identified and that any answers you provided or discussed during the interview will have no bearing on your standing within your organisation or community. None of your personal information will be kept.**

**Do you consent to the information we have discussed being included in this study? Y/N**

**THANK YOU FOR GIVING YOUR CONSENT.**

**If there is anything that we have discussed today that you would like to seek help with, or discuss with a professional, please remember that there are many counselling services available, such as Lifeline (Tel; 13 1 1 14), or Beyond Blue (Tel; 1300 224 636), or Mensline Australia (Tel;1300 78 99 78) or 1300 DRIVER Hotline (Tel; 1300 374 837)).**

**If you have any further questions regarding this study, please feel free to contact me via my email address (22028151@student.westernsydney.edu.au).**

**In addition, if you would like a copy of this interview transcript and/or a de-identified data summary and/or a copy of the research findings please contact me via my email address (22028151@student.westernsydney.edu.au). As a reminder, your contact details will be deleted on delivery of the documents.**

**Thank you for your participation in this study—this concludes our interview today. I will now turn off the recording device.**

## Appendix C. Interview Schedule—Transport Managers

*(Interviewer instructions/reminder) Each interview will last between 30 to 60 min depending on the elaboration of the responses given. Spontaneous or prompt questions not listed in this schedule may be asked to encourage narrative and to ensure responses have been fully understood by the interviewer. The interview questions ask separate questions in respect of Heavy Vehicle National Law (HVNL) jurisdictions and Workplace Health and Safety (WHS) jurisdictions as identified throughout. Italicised questions are to guide the interviewer.*

**Thank you for your time today. The purpose of this interview is to gather information regarding your beliefs and attitudes towards truck driver fatigue and its management. Just a reminder, that everything you say is completely confidential and you will not be identified in any way. You are free to withdraw from this interview at any time.**

**Do you give your consent to participate in this study? Y/N**

**Do you give your consent for me to proceed with the recording? Y/N**

1. Demographics and Experience

1.1. How old are you?

1.2. Do you identify as Male/Female/Other?

1.3. Were you born in Australia? *(What country were you born in? What is your cultural heritage? Is English your first language? Do you identify as indigenous Aboriginal or TSI?)*

1.4. What is your highest level of education?

1.5. How long have you been managing truck drivers?

1.6. Have you done driving work in the past? (How long? What kind of driving work?)

1.7. Have you had any breaks in your career where you worked in a job outside the transport industry? If so, when were these breaks and how long did you spend the other role/s?

1.8. What kinds of transport companies have you worked for? (small operators/major companies, etc.)

1.9. What kind of work are you managing now? *For example, general freight, refrigerated produce,* etc.*?*

1.10. Are you currently an employed manager or self-employed?

*(To identify different experiences between employed managers and owner/managers.)*

1.11. Are the drivers you manage currently paid per hour, per trip, per km, *(or main method)* etc.*?*

2. Learning about truck driver fatigue and fatigue management

2.1. Are you aware of the physical effects of truck driver fatigue? Do you think most managers can describe them? *(To identify the extent of managers/controllers existing knowledge about the physical effects of truck driver fatigue.)*

***If the participant answers ‘Yes’ continue from Q2.2 to Q2.4 only. If the participant answers ‘No’, skip to Q 2.5.***

2.2. How do you think most managers/controllers learn about the **physical effects of fatigue on truck drivers?** *For example, training courses, internet, friends, colleagues, managers/controllers, trade unions or associations, self-taught/research, other sources? (To identify where they sourced their information about the physical effects of truck driver fatigue and to assess the breadth of their information sources.)*

2.3. How do you think most managers/controllers learn about **the laws regarding truck driver fatigue?** *For example, training courses, internet, friends, colleagues, managers/controllers, trade unions or associations, self-taught/research, other sources? (To identify where they sourced their information about the laws regarding truck driver fatigue laws and to assess the breadth of their information sources.)*

2.4. How do you think most managers/controllers learn about **company rules and operational guidelines regarding truck driver fatigue management?** *For example, training courses, internet, friends, colleagues, managers/controllers, self-taught/research, other sources? (To identify where they sourced their knowledge about company rules regarding truck driver fatigue and to assess the breadth of their information sources.)*

***If the participant has answered ‘No’ to Q 2.1, add the following preamble relevant to the participant’s jurisdiction:***

***(In HVNL jurisdictions,*** Heavy Vehicle National Law describes fatigue as ***”including but not being limited to feeling sleepy, feeling physically or mentally tired, weary, or drowsy and feeling exhausted or lacking energy and behaving in a way consistent with any or all these experiences”.)***

(***For WHS jurisdictions***, Safework Australia defines fatigue as ***”…. a state of mental and/or physical exhaustion that reduces a person’s ability to perform work safely and effectively”.)***

2.5. Having described the definition of fatigue, do you think this is something most managers/controllers are aware of? *(To identify their perception of fatigue in relation to truck drivers.)*

**Coding Schema—Interview Items and TPB Components**

3. Beliefs, attitude—RQ 2—How these shape compliance/non-compliance

*Belief: ”Something that is accepted, considered to be true” (Merriam-Webster, 2022)* [91].

*Attitude: ”a disposition to respond favourably or unfavourably to an object, person, institution or event’ that derives reasonably from accessible beliefs about the behaviour”. Beliefs and attitudes are subject to change (Ajzen, p3, p30, 137)* [36].

***(If participants answered ‘Yes’ to Q 2.1, ask the series of questions marked with ‘a’. If participants answered ‘No’ to Q 2.1, ask the series of questions marked with ‘b’.)***

**_ _ _ _**

3.1.a. What do you think/believe are the main signs of fatigue? (To identify their understanding of the definition of fatigue relevant to research and legislative definitions.)

3.2.a. How do you think/believe most truck drivers experience it? *For example, feeling sleepy, struggling to stay awake, yawning, can’t concentrate, feeling exhausted or lacking energy, etc. (This is for me to compare their understanding of driver fatigue in relation to the formalised definition/s of fatigue for later use in my discussion chapter.)*

3.3.a. In your opinion, is driver fatigue an important issue for managers/controllers? *(To ascertain their belief about the importance of truck driver fatigue as an issue for their cohort.)*

3.4.a. In your opinion, do **the physical effect of truck driver fatigue** affect truck drivers’ ability to do their job properly? *(To identify their beliefs about the significance of the physical effects on truck driver fatigue.)*

3.5.a. Could you give me an example of how you think/believe driver fatigue could increase risks for truck drivers? *(To ascertain their capacity to identify risks regarding truck driver fatigue.)*

3.6.a. In your opinion, what are most truck driver managers’/controllers’ attitudes towards **truck driver fatigue laws?** *For example, are they effective in helping managers/controllers manage the fatigue of their truck drivers? Are they easy or difficult to comply with? Do they help managers/controllers and truck drivers get the job done? (To ascertain their attitudes towards truck driver fatigue laws, perceptions of effectiveness and how the laws affect their work.)*

3.7.a. In your opinion, what are most truck driver managers’/controllers’ attitudes towards **company rules** and driver fatigue? *For example, do they think they are effective in helping truck drivers manage their fatigue? Do they think they are easy or difficult to comply with? Do they help truck drivers get the job done? (To ascertain their attitudes towards company rules regarding truck driver fatigue, perceptions of effectiveness and how they affect their work.)*

3.8.a. In your opinion, how important is it for managers/controllers to ensure their truck drivers comply with **truck driver fatigue laws**? *(To assess their own beliefs about the importance of compliance with truck driver fatigue laws.)*

3.9.a. In your opinion, how important is it for managers/controllers to ensure their drivers comply with **company rules regarding driver fatigue**? *(To assess their own beliefs about the importance of compliance with company rules regarding driver fatigue.)*

3.10.a. Thinking about truck driver fatigue and the laws and company rules about it, what information source do you trust the most? Why? *(Relates to questions 2.2, 2.3 and 2.4; to assess where they sourced their most trusted information about truck driver fatigue.)*

3.11.a. In your opinion, do most companies manage truck driver fatigue in an effective way? What main strategies (if any) do they use to manage driver fatigue? *For example, do they offer training programs? Do they distribute truck driver fatigue-related advertising material, inter-company emails,* etc.*? Is fatigue mentioned during induction programs? (In WHS jurisdictions, do managers/controllers receive any training or induction information? Are they informed about driver fatigue management plans,* etc.*)?* In your opinion, are these strategies effective? Why/why not? Is there anything you think/believe could be done better? *(To assess beliefs about how truck driver fatigue is managed.)*

3.12.a. Is there one strategy that stands out as being really helpful to you? *For example, what do you think the company does best?* Is there anything you think/believe could be done better?

*For example, is there one thing that stands out as being unhelpful or a waste of time*? *(To assess beliefs about how truck driver fatigue is managed.)*

3.13.a. Having now discussed truck driver fatigue, in your opinion, how do most managers/controllers think/believe truck driver fatigue is a priority/safety issue? *For example, on a scale of 1 to 10 where 1 is the least important and 10 is the most important, how would they rate truck driver fatigue as a priority issue?* Why? *(To assess beliefs about how managers/controllers perceive the importance of truck driver fatigue.)*

3.14.a. Having now discussed driver fatigue in relation to rules and regulations, what do you see as the main things that contribute to truck driver fatigue? *(To assess their own belief about causes of truck driver fatigue.)*

3.15.a. Are there ways to manage it better than it is managed now? If yes, how? *(To assess their own belief about solutions regarding truck driver fatigue.)*

**_ _ _ _**

***Skip alternative to Q 3.1a as it is not relevant to participants who answer ‘No’ to Q 2.1.***

***Skip alternative to Q 3.2a as it is not relevant to participants who answer ‘No’ to Q 2.1.***

3.3.b. Having defined fatigue, do think/believe it is an important issue for managers/controllers? *(To ascertain their belief about the importance of truck driver fatigue as an issue for them and their cohort.)*

3.4.b. Having defined fatigue, do you think/believe **the physical effect of truck driver fatigue** could affect truck drivers’ ability to do the job properly? *(To identify their beliefs about the significance of the physical effects of truck driver fatigue.)*

3.5.b. Could you give me an example of how you think/believe driver fatigue could increase risks for truck drivers? *(To ascertain their capacity to identify risks regarding truck driver fatigue.)*

3.6.b. Having defined fatigue, how do you think/believe **truck driver fatigue laws** might affect truck driver managers/controllers? *For example, could they be effective in helping managers/controllers manage the fatigue of their truck drivers? Are they easy or difficult to comply with? Could they help managers/controllers and truck drivers get the job done?? (To ascertain their attitudes towards truck driver fatigue laws, perceptions of effectiveness and how the laws affect their work.)*

3.7.b. Having defined fatigue, how do you think/believe **company rules** might affect truck drivers? *For example, could they be effective in helping truck drivers manage their fatigue? Are they easy or difficult to comply with? Could they help truck drivers get the job done? (To ascertain their attitudes towards company rules regarding truck driver fatigue, perceptions of effectiveness and how these rules may affect their work.)*

3.8.b. Having defined fatigue, in your opinion, how important is it for managers/controllers to ensure their truck drivers comply with **truck driver fatigue laws**? *(To assess their own beliefs about the importance of compliance with truck driver fatigue laws.)*

3.9.b. Having defined fatigue, in your opinion, how important is it for managers/controllers to ensure their truck drivers comply with **company rules regarding truck driver fatigue**? *(To assess their own beliefs about the importance of compliance with company rules regarding truck driver fatigue.)*

***Skip alternative to Q 3.10a as it is not relevant to participants who answer ‘No’ to Q 2.1.***

3.11.b. If other managers/controllers are unaware of driver fatigue, why do you think they are not aware? *For example, do other companies not offer training programs? Do they not distribute truck driver fatigue related advertising material or inter-company emails* etc.*? Is fatigue not mentioned during induction programs? (In WHS jurisdictions, do managers/controllers not receive any training or induction information? Are they not informed about driver fatigue management plans,* etc.*)?* What do you think/believe could be done better? *(To assess beliefs about how truck driver fatigue is managed.)*

***Skip alternative to Q 3.12a as it is not relevant to participants who answer ‘No’ to Q 2.1.***

***Skip alternative to Q 3.13a as it is not relevant to participants who answer ‘No’ to Q 2.1.***

3.14.b. Having now discussed driver fatigue in relation to rules and regulations, what do you see as the main things that contribute to truck driver fatigue? *(To assess their own belief about causes of truck driver fatigue.)*

3.15.b. Having defined fatigue, do you think there are ways to manage it better than it is managed now? If yes, how? *(To assess their own belief about solutions regarding truck driver fatigue.)*

4. Subjective norms—RQ 3—Key Influences on compliance/non-compliance

*(In accordance with the Theory of Planned Behaviour, for certain cohorts and professions, there may be a fine line between subjective norms and perceived behavioural controls—for law enforcement and policing practices this is important to consider and needs careful unpacking in the interviews and related questions).*

***(If participants answered ‘Yes’ to Q 2.1, ask the series of questions marked with ‘a’. If participants answered ‘No’ to Q 2.1, ask the series of questions marked with ‘b’.)***

4.1.a. Is truck driver fatigue something that is discussed in most transport company workplaces or amongst your industry colleagues who manage truck drivers? How often is it discussed? What triggers these discussions? *For example, a crash, a fine being issued to a company,* etc.*? (To ascertain potential origins and recency of subjective norms regarding truck driver fatigue.)*

4.2.a. What do you think/believe could influence how other managers/controllers perceptions of **truck driver fatigue**? *For example, could it be the opinion or personal experiences of other managers/controllers, or reported experiences of truck drivers—some of their truck drivers might report experiences of fatigue or experiences of long periods of driving without feeling fatigued? How do you think/believe this might influence manager/controllers’ perceptions of the physical effects of fatigue? (To identify the influences of the co-worker cohort about the physical effects of truck driver fatigue.)*

4.3.a. In your opinion, what are the strongest influences on the way managers/controllers think about **compliance with truck driver fatigue management requirements**? *For example, are managers/controllers more influenced by company rules or fatigue management laws or are these equally influential? Why/why not? Do the shared opinions of managers/controllers influence each other’s responses to truck driver fatigue? (To identify the influences within managers’/controllers’ workplaces regarding truck driver fatigue laws and compliance.)*

4.4.a. Do you think/believe managers/controllers are influenced about compliance issues by other factors relating to driver fatigue, such as things that may heighten their awareness of it? *For example, their awareness of road crashes, injury, death,* etc., *due to driver fatigue?* Do you think/believe these types of factors influence their compliance or non-compliance? Are there any other things or influences that may affect manager/controllers’ compliance or non-compliance with driver fatigue rules and regulations, such as pressure from customers? *(To identify their understanding of other influences on managers/controllers and how they manage truck driver fatigue.)*

4.5.a. Whilst managers/controllers are themselves generally influenced by senior managers and the operational guidelines of the company in terms of workplace rules and practices, who or what do you think/believe would be other influences that may affect their compliance or non-compliance with driver fatigue laws and company rules? *For example, friends, family, partners, financial concerns,* etc.*? (To identify the influence of significant others, for example friends, family, partners, financial concerns,* etc.*, on managers/controllers’ compliance/non-compliance and attitudes/beliefs regarding truck driver fatigue laws and company rules.)*

**_ _ _ _**

***Skip alternative to Q 4.1a as it is not relevant to participants who answer ‘No’ to Q 2.1.***

4.2.b. Having defined fatigue, what do you think/believe could influence how other managers/controllers perceive **truck driver fatigue**? *For example, could it be the opinion or personal experiences of other managers/controllers, or reported experiences of truck drivers—some of their truck drivers might report experiences of fatigue or experiences of long periods of driving without feeling fatigued? How do you think/believe this might influence manager/controllers’ perceptions of the physical effects of fatigue? (To identify the influences of the co-worker cohort about the physical effects of truck driver fatigue.)*

4.3.b. Having defined fatigue, in your opinion, what might be the strongest influences on the way managers/controllers think about **compliance with truck driver fatigue management requirements**? *For example, are managers/controllers more influenced by company rules or fatigue management laws or are these equally influential? Why/why not? Do the shared opinions of managers/controllers influence each other’s responses to truck driver fatigue? (To identify the influences within managers’/controllers’ workplaces regarding truck driver fatigue laws and compliance.)*

4.4.b. Having defined driver fatigue, do you think/believe managers/controllers could be influenced by other factors relating to driver fatigue, such as things that may heighten their awareness of it, to increase compliance? *For example, their awareness of road crashes, injury, death,* etc., *due to driver fatigue?* Do you think/believe these types of factors could influence their compliance or non-compliance? Are there any other things or influences that may affect truck drivers’ compliance or non-compliance with driver fatigue rules and regulations, such as pressure from customers*? (To identify their understanding of other influences on truck drivers and truck driver fatigue.)*

4.5.b. Whilst managers/controllers are themselves generally influenced by senior managers and the operational guidelines of the company in terms of workplace rules and practices, who or what do you think/believe would be other influences that may affect their compliance or non-compliance with driver fatigue laws and company rules? *For example, friends, family, partners, financial concerns* etc.*? (To identify the influence of significant others, for example friends, family, partners, financial concerns,* etc.*, on managers’/controllers’ compliance/non-compliance and attitudes/beliefs regarding truck driver fatigue laws and company rules)*

5. **Perceptions of behavioural control—RQ 4—*How easy/difficult it is to comply/not comply with the behaviour (compliance/non-compliance with driver fatigue rules/regulations/laws/guidelines)***

***All questions apply regardless of the participants answer to Q 2.1.***

5.1. Having discussed and identified truck driver fatigue, in your opinion, when a manager/controller starts their shift do you think/believe most of them intend to comply with **truck driver fatigue laws**? *(To ascertain initial short-term intentions regarding compliance or non-compliance with truck driver fatigue laws.)*

5.2. Having discussed and identified truck driver fatigue, in your opinion, when a manager/controller starts their shift do you think/believe most of them intend to comply with **company rules** about truck driver fatigue? *(To ascertain initial short-term intentions regarding company rules about truck driver fatigue.)*

5.3. In your opinion, what kind of things might change a manager/controller’s intentions about how they manage truck driver fatigue? *For example, if there is a delay, how might that influence their intentions to facilitate compliance with truck driver fatigue laws and/or company rules?* Can you give me some examples of the types of barriers that may disrupt a manager/controller’s intentions and behaviours regarding facilitating truck driver fatigue compliance? What is it that would make the controller’s management of this situation easy or difficult? *(To ascertain triggers that may affect the perception of behavioural control, intentions and behaviour of managers’/controllers’ management of truck driver fatigue compliance.)*

5.4. When drivers are on the road, and now thinking about this in context, how easy or difficult do you think/believe it would be for truck drivers to ignore rest requirements? *For example, would it be easy or difficult for them to ignore the requirements of laws and/or company rules regarding truck driver fatigue? Could they ignore instructions from their manager/controller? (To identify perceptions of behavioural control managers/controllers have over truck drivers whilst the drivers are on the road.)*

5.5. When finishing their shift, if drivers report to managers/controllers, in your opinion, how easy or difficult would it be for truck drivers to hide their compliance or non-compliance with driver fatigue laws and company rules? If managers/controllers are aware that drivers have not complied with driver fatigue laws and company rules, in your opinion, how easy or difficult might it be for them to overlook this? *(To ascertain how easy or difficult it is in reality, for managers/controllers to ignore issues of compliance/non-compliance with laws and company rules regarding truck driver fatigue.)*

5.6. At the start of their career, how easy or difficult do you think/believe it would be for managers/controllers to ignore rules and regulations? Would this become easier or more difficult over time? *For example, does it become easier or more difficult to follow laws and company rules starting out, or mid-career or towards the end of their career? Is it easier or more difficult for managers/controllers with more or less experience to comply with/follow/uphold driver fatigue laws and company rules? (To ascertain ease or difficulty of compliance with and management of truck driver fatigue laws and company rules at different stages of their careers.)*

5.7. Having discussed how easy or difficult compliance or non-compliance would be, what do you think/believe would be the key thing that would shape a manager’s/controller’s intention to comply? *For example, increased knowledge and experience? Achieving a higher position in the company? (To identify temporal/other factors that may increase ease or difficulty of compliance/non-compliance as well as to identify key influences on intention.)*

5.8. Having discussed and identified truck driver fatigue, in your opinion, when thinking about compliance/non-compliance and driver fatigue management, do most managers/controllers think about law enforcement of this issue, and do you think enforcement influences their intention to comply or not comply with driver fatigue laws? *For example, is awareness of law enforcement involvement in this issue likely to make managers/controllers think about ensuring their truck drivers take rest breaks? (To ascertain the perceived behavioural controls that may encourage a manager/controller to think more about truck driver fatigue and compliance or non-compliance with truck driver fatigue laws—this again is about how intention is shaped.)*

5.9. In your opinion, how easy or difficult do current law enforcement practices make it for managers/controllers to implement/train/remind drivers to comply, and to themselves comply, with the laws regarding truck driver fatigue? Why? What do you think/believe enforcement might mean for managers/controllers in terms of the law if they do not comply? *For example, fines, arrest, other outcomes, etc*.? *(To ascertain, in the context of perceptions of enforcement practices, the extent of control these perceptions have on managers’/controllers’ intentions and behaviour regarding compliance or non-compliance with truck driver fatigue laws and company rules, and whether the awareness of law enforcement shapes their workplace practices.)*

5.10. In your opinion, what do you think/believe would be the main thing that shapes the overall intention of the managers/controllers regarding compliance with truck driver fatigue laws and company rules? *For example, a desire to comply with the law, a desire to comply with company rules, safety concerns, customer pressures, instructions/pressure from senior managers,* etc.*? (To ascertain the most significant influence that shapes the intention of managers/controllers to uphold laws and company rules regarding truck driver fatigue.)*

**Thank you for your time today in participating in this interview. Please be assured that all the data you have supplied will be de-identified and that any answers you provided or discussed during the interview will have no bearing on your standing within your organisation or community. None of your personal information will be kept.**

**Do you consent to the information we have discussed being included in this study? Y/N**

**THANK YOU FOR GIVING YOUR CONSENT.**

**If there is anything that we have discussed today that you would like to seek help with, or discuss with a professional, please remember that there are many counselling services available, such as Lifeline (Tel; 13 1 1 14), or Beyond Blue (Tel; 1300 224 636), or Mensline Australia (Tel;1300 78 99 78) or 1300 DRIVER Hotline (Tel; 1300 374 837)).**

**If you have any further questions regarding this study, please feel free to contact me via my email address (22028151@student.westernsydney.edu.au).**

**In addition, if you would like a copy of this interview transcript and/or a de-identified data summary and/or a copy of the research findings please contact me via my email address (22028151@student.westernsydney.edu.au). As a reminder, your contact details will be deleted on delivery of the documents.**

**Thank you for your participation in this study—this concludes our interview today. I will now turn off the recording device.**
